# Searching for Novel HDAC6/Hsp90 Dual Inhibitors with Anti-Prostate Cancer Activity: In Silico Screening and In Vitro Evaluation

**DOI:** 10.3390/ph17081072

**Published:** 2024-08-15

**Authors:** Luca Pinzi, Silvia Belluti, Isabella Piccinini, Carol Imbriano, Giulio Rastelli

**Affiliations:** Department of Life Sciences, University of Modena and Reggio Emilia, Via Giuseppe Campi 103, 41125 Modena, Italy; luca.pinzi@unimore.it (L.P.); silvia.belluti@unimore.it (S.B.); isabella.piccinini@unimore.it (I.P.); carol.imbriano@unimore.it (C.I.)

**Keywords:** HDAC6, Hsp90, drug design, virtual screening, prostate cancer

## Abstract

Prostate cancer (PCA) is one of the most prevalent types of male cancers. While current treatments for early-stage PCA are available, their efficacy is limited in advanced PCA, mainly due to drug resistance or low efficacy. In this context, novel valuable therapeutic opportunities may arise from the combined inhibition of histone deacetylase 6 (HDAC6) and heat shock protein 90 (Hsp90). These targets are mutually involved in the regulation of several processes in cancer cells, and their inhibition is demonstrated to provide synergistic effects against PCA. On these premises, we performed an extensive in silico virtual screening campaign on commercial compounds in search of dual inhibitors of HDAC6 and Hsp90. In vitro tests against recombinant enzymes and PCA cells with different levels of aggressiveness allowed the identification of a subset of compounds with inhibitory activity against HDAC6 and antiproliferative effects towards LNCaP and PC-3 cells. None of the candidates showed appreciable Hsp90 inhibition. However, the discovered compounds have low molecular weight and a chemical structure similar to that of potent Hsp90 blockers. This provides an opportunity for structural and medicinal chemistry optimization in order to obtain HDAC6/Hsp90 dual modulators with antiproliferative effects against prostate cancer. These findings were discussed in detail in the study.

## 1. Introduction

Histone deacetylases (HDACs) are a family of proteins primarily responsible for regulating the acetylation status of lysine residues in histone tails, thus acting as key epigenetic modulators in several biological processes [1,2]. HDACs are classified into four major classes: class I (HDAC1, 2, 3, 8), class II, divided into subgroups IIa (HDAC4, 5, 7, 9) and IIb (HDAC6, 10), class III (sirtuins 1–7), and class IV (HDAC11) [3]. Classes I, II, and IV require Zn^2+^ for catalysis, while sirtuins employ Nicotinamide Adenine Dinucleotide (NAD) as a cofactor [4]. Recent studies have shown that several Zn-dependent HDACs, and especially class I and class II isoforms, are overexpressed in various tumors like breast, prostate, and liver cancers, multiple myeloma and neuroblastoma, making these enzymes suitable targets for the development of novel therapeutic agents [5]. Among those, HDAC6 is extensively studied due to its involvement in diverse biological and pathological pathways, making it of therapeutic relevance in cancer, neurodegenerative disorders [6], and other diseases [7]. In addition, several studies have demonstrated that HDAC6 overexpression stimulates cell motility and promotes endothelial cell migration, triggering metastasis and angiogenesis [8]. Moreover, HDAC6 is regulated by estrogen activity; hence, its activity might be involved in estrogen receptor (ER)-positive breast cancer [9]. Given the wealth of applications and therapeutic relevance of this target, there is considerable interest in developing inhibitors of this enzyme [10,11,12].

The design of novel HDAC modulators primarily exploits the coordination with the Zn^2+^ ion in the active site of the enzymes. This binding mechanism has enabled the development of potent HDAC inhibitors, although they often lack selectivity among various histone deacetylase isoforms [13]. The pharmacophoric model of HDAC inhibitors (HDACis) includes: (i) a cap group interacting with the surface of the enzyme catalytic cavity; (ii) a linker fitting into the catalytic tunnel, and; (iii) a metal-binding group (i.e., zinc-binding group, ZBG), coordinating the catalytic zinc ion [14]. Despite the discovery of potent HDAC inhibitors, such as Belinostat, Panobinostat and Vorinostat [15], pan-HDACis face several clinical issues mainly due to low selectivity, which often results in side effects that limit their therapeutic potential [16,17,18].

Furthermore, several studies reported that HDAC inhibitors may have limited therapeutic effects on solid cancers, probably due to their low efficiency in reaching the tumor site [19]. Single-target approaches based on HDACis often lead to temporary effects, while combining HDAC inhibitors with other therapeutic agents targeting different pathways exhibits more effective and durable therapeutic responses [16,17,18]. In particular, HDAC6 regulates a number of substrates, including heat shock protein 90 (Hsp90), which is a chaperone protein involved in the correct folding of many proteins, some of them being cancer-relevant targets [20]. In cancer cells, Hsp90 is responsible for uncontrolled proliferation and apoptotic resistance by means of the regulation of several so-called clients, including steroid hormone receptors, kinases (e.g., Akt, Raf-1, Bcr-Abl, Cdk4, and Wee1), histone deacetylases (i.e., HDAC1 and HDAC6), and other oncogenic proteins [21,22]. Hsp90 is often overexpressed in several cancers [23,24,25,26], making it a suitable target for anticancer drug discovery. However, despite the discovery of a number of highly active drug candidates that modulate this enzyme, results from clinical trials on Hsp90 inhibitors were disappointing, mainly because of undesired adverse effects [25]. In addition, monotherapies with Hsp90 inhibitors have been demonstrated to provide acquired resistance in different types of cancer and hepatotoxicity issues [27,28], which have hampered their approval for clinical use [29]. Rather, strategies based on the combination of Hsp90 inhibitors with other anticancer drugs have shown promising results [15,30,31,32]. Indeed, HDAC6 interacts with Hsp90 in several ways [15]. Moreover, HDAC6 is one of the most studied clients of Hsp90 [33]. In addition, HDAC6 can reversibly modulate Hsp90 activity through post-translational modifications, regulating its acetylation levels and disrupting its chaperone function [34,35,36].

Based on these findings, a multi-target approach aiming to inhibit both Hsp90 and HDAC6 with a single molecular entity represents a promising strategy against several types of cancers. In line with the polypharmacology concept [37], this approach could reduce tumor cell viability by interfering with different cancer-relevant biological pathways and help to overcome issues, such as drug resistance and toxicity concerns [38,39]. In advanced-stage PCA, the role of HDAC6 in regulating androgen receptor (AR) hypersensitivity, occurring mainly through Hsp90 acetylation/deacetylation [40], underscores the therapeutic potential of dual HDAC6/Hsp90 inhibitors. Although both targets were studied from a structural perspective, the reported number of dual binders is limited and is primarily characterized by poor drug-like properties, such as high molecular weight and complex chimeric structures [15,41]. This is mainly due to the fact that HDAC6 and Hsp90 belong to different protein families and present two substantially different binding pockets.

In this context, novel valuable opportunities may arise from the application of de novo design and virtual screening on large libraries of small molecules, as these approaches can help in discovering novel chemotypes with a defined multi-target activity profile [42,43,44]. On these premises, we performed a series of ligand- and structure-based in silico screenings of commercially available compounds, followed by experimental validation with recombinant enzymes and in PCA cells. In particular, substructure searches within the ZINC database [45], followed by 2D similarity estimations and docking calculations into selected conformations of both targets were performed. The drug-like properties of the resulting compounds were carefully investigated. These analyses led to the selection of a series of compounds, which were experimentally tested in vitro for their inhibition ability against Hsp90 and HDAC6, and for their antiproliferative activity in PC-3 and LNCaP PCA cell lines. While we obtained potent inhibitors of HDAC6 with antiproliferative activity, the compounds did not inhibit Hsp90. Further investigation into the binding mode of these compounds and a comparison of their scaffold with already reported Hsp90 inhibitors revealed that small changes in their chemical structures should be sufficient to achieve dual HDAC6/Hsp90 inhibition.

Altogether, the performed analyses, along with the relatively low-decorated scaffold of the top candidates identified in the screening, provide the opportunity for chemical optimization in order to obtain the desired, structurally novel, drug-like HDAC6/Hsp90 dual inhibitors.

## 2. Results

### 2.1. Virtual Screening and Selection of Commercially-Available Dual Inhibitor Candidates

The ZINC database (over 120 million purchasable “drug-like” compounds) [45] was used as a starting point to investigate the presence of the commercially available compounds of interest. According to the literature data, compounds modulating Hsp90 and HDAC6 activity usually possess chemical moieties able to establish specific interactions with hot-spot residues in the binding site of the two targets [15,32]. In particular, a ZBG is required for a molecule to be active on HDAC6 [46], while functional groups able to establish H-bond interactions with D93 and the conserved network of water molecules are needed for Hsp90 inhibitory activity [47].

To identify molecules potentially endowed with dual inhibitory activity, extensive substructure filtering was performed on the screening compounds. This process utilized HDAC6 and Hsp90 warheads identified in our previous work [15,32], along with a series of additional moieties reported more recently, for a total of 10 different ZBGs (HDAC6) and 17 D93-binding groups (Hsp90) (see Figure S1). HDAC6 warheads included hydroxamic acid (HA) [46], mercaptoacetamide [48], and trifluoromethyloxadiazole (TFMO), which we explored in previous studies for the development of HDAC6 inhibitors [32,49]. Additionally, less explored moieties, such as *N*-(2-aminophenyl)acetamide, *N*-(2-hydroxyphenyl)acetamide, *N*-(2-mercaptophenyl)acetamide, 1-mercaptoacetone, (sulphamoylamino)methane, ethylboronic acid, 3-methoxy-1*H*-pyridine-2-thione, and 1-methoxypyridine-2-thione were considered [46]. For Hsp90, besides the well-known Hsp90 warheads used in reference [32], additional less-explored D93-binding moieties were included to increase the chances of identifying a suitable hit with dual activity.

These analyses resulted in 182,392 compounds containing at least one of the selected ZBGs, 1,388,699 molecules bearing a D93-binding group, and only 598 compounds containing both of them. Given the significant reduction in compounds fulfilling both requisites, we decided to continue investigating compounds with a single requirement. These compounds were analyzed through extensive 2D fingerprints-based similarity estimations with respect to known inhibitors of the two targets collected from ChEMBL. This allowed us to include compounds that, although bearing only one of the identified warheads, are structurally similar to known HDAC6 and Hsp90 inhibitors, thereby being closer to the HDAC6 and Hsp90 chemical space. We performed the 2D similarity profiling using the MACCS and ECFP4 molecular fingerprints [50,51], with settings described in the Methods section. Only compounds that were similar to at least one of the Hsp90 or HDAC6 queries, according to commonly accepted similarity thresholds (i.e., MACCS Tanimoto score ≥ 0.8 and ECFP4 Tanimoto score ≥ 0.3) [50], were selected for further investigation in the virtual screening campaign, resulting in a total of 43,216 molecules. These compounds were subsequently docked into selected conformations of Hsp90 and HDAC6, as reported in Methods. We selected seven crystallographic structures of Hsp90 and two of HDAC6 to perform docking calculations, in order to take into account their binding site flexibility. Moreover, these structures also allowed the consideration of different types of zinc coordination that HDAC6 inhibitors can exhibit with the catalytic zinc in the protein binding site.

**Figure 1:**
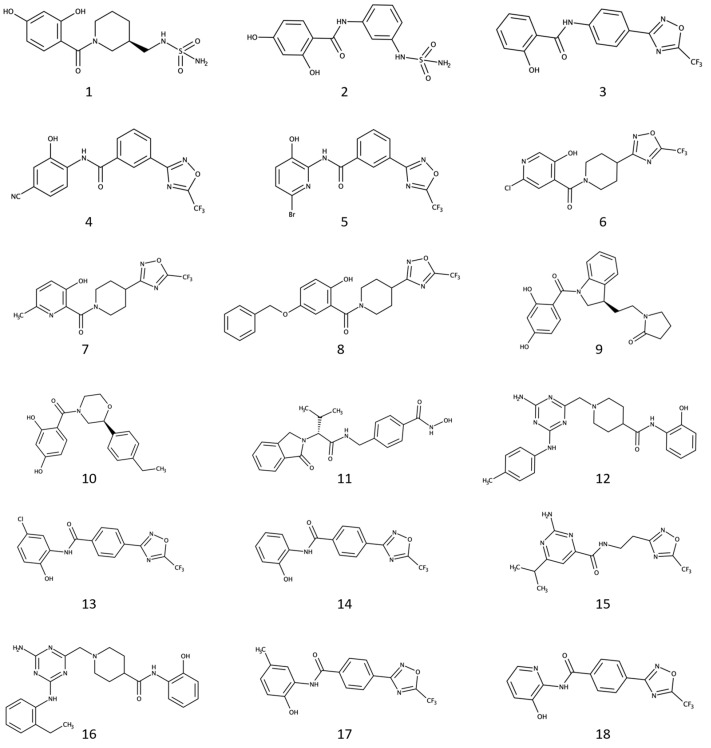
Chemical structures of the 18 commercially available compounds selected from the virtual screening campaign.

The selection process, which took into account: (i) the binding scores and poses predicted for the compounds in the two targets; (ii) the chemical similarities of the compounds with reported inhibitors of Hsp90 and HDAC6, and; (iii) the drug-like properties of the compounds as evaluated by *QikProp* (Schrödinger Suite 2020-1) (Table S1) [52], led to 18 particularly interesting dual inhibitor candidates (Figure 1).

Of note, 16 out of the 18 compounds bear one of the selected metal-binding groups (i.e., all the selected molecules, excluding **9** and **10**), while only six showed D93-binding moieties (i.e., candidates **1**, **2**, **9**, **10**, **15**, and **16**). These results might be attributed to the fact that: (i) compounds with the structural features necessary for the efficient binding on such different targets are poorly represented in the chemical space covered by the ZINC database, and; (ii) the design of potent HDAC6 inhibitors might require a higher number of structural constraints to be satisfied compared to Hsp90 modulators. Considering that most of the selected ligands bear an established ZBG, compounds were initially tested against HDAC6 activity in vitro. Afterward, compounds with IC_50_ values below 20 µM were tested for Hsp90 inhibition and assessed for their antiproliferative activity on LNCaP and PC-3 prostate cancer cells.

### 2.2. Four Selected Ligands Inhibit HDAC6 and Decrease Viability of Human PCA Cells

The 18 selected compounds were initially tested in vitro against the HDAC6 recombinant protein, following the procedure described in the Methods section. Although many of the selected candidates exhibited no or low (percentage of inhibition lower than 50% at 20 µM) HDAC6 inhibitory activity, compounds **4**, **8**, **11**, and **18** inhibited HDAC6 activity with IC_50_ concentrations in the low micromolar or nanomolar ranges (Table 1). These results guided the selection of these four compounds for further evaluation of Hsp90 inhibitory activity. Unfortunately, none of the four compounds resulted in being effective at inhibiting Hsp90 in vitro. These results might derive from the absence of functional groups on compounds **4**, **8**, **11**, and **18** that efficiently establish interactions with the conserved waters and the key residue D93 present in the Hsp90 binding site [28], albeit having resulted to be similar to recognized Hsp90 warheads. For example, compounds **4**, **8**, and **18** embed hydroxyl phenol and hydroxyl pyridine ring moieties (Figure 1) similar to the isopropyl-resorcinol warhead present in potent Hsp90 blockers [28], and also on Hsp90/HDAC6 dual inhibitors (e.g., C.17) [41]. This suggests that substituting these chemical moieties with ones that bind more efficiently to the Hsp90 binding site might result in the desired dual inhibitory activity.

To investigate the potential anti-tumor activity of the four compounds that displayed HDAC6-inhibitory activity (Table 1), we tested their antiproliferative ability in LNCaP and PC-3 cells. LNCaP is an androgen-responsive prostate adenocarcinoma cell line isolated from a lymph node metastatic lesion [53], while the PC-3 cell line derives from a prostate cancer bone metastasis and exhibits androgen-independent growth, representing a more aggressive, castration-resistant prostate cancer model [54]. Dose–response assays were performed with the candidate compounds, and cell viability assays were performed after 72 h, as detailed in the Methods section. GI_50_ values were determined as the concentrations that inhibit cell growth by 50% following 72 h of treatment (Table 1). All candidate compounds decrease the proliferation of PCA cells. Compound **4** was particularly effective, while compound **8** exhibited the lowest antiproliferative activity in both PC-3 and LNCaP cells, with a GI_50_ of about 50 µM. Compounds **11** and **18**, on the other hand, demonstrated higher antiproliferative activity on PC-3 cells, which is interesting given that these cells are considered a representative model for tumors more aggressive and resistant to first-line hormone therapy.

### 2.3. HDAC6-Targeting in LNCaP and PC-3 Cells

**Figure 2:**
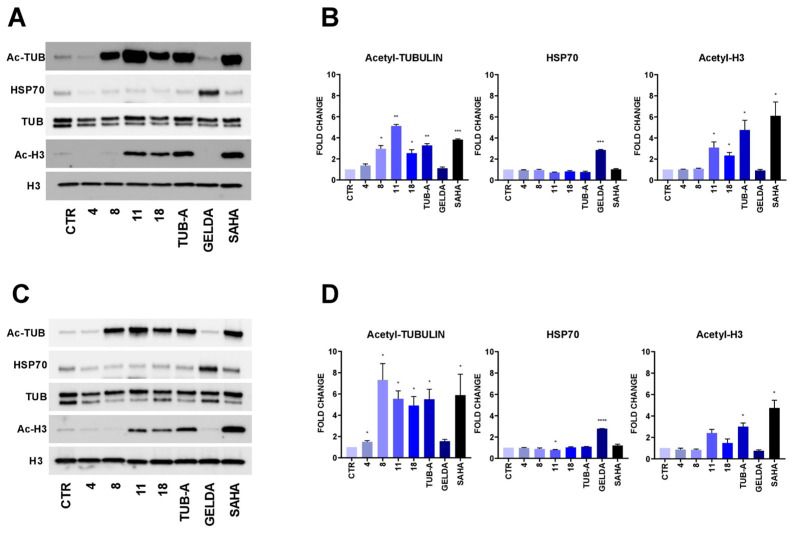
Western blot analysis and relative quantification of the expression levels of acetylated-Tubulin (Ac-TUB), acetylated H3 (Ac-H3), and HSP70 in LNCaP (**A**,**B**) and PC-3 (**C**,**D**) cells treated for 24 h at GI_50_ concentration. Tubastatin-A was used as HDAC6-specific positive control, Geldanamycin (GELDA) as HSP90-specific positive control, and SAHA as pan-HDAC inhibitor. Protein expression was normalized with Tubulin levels (Ac-TUB and HSP70) or total H3 (Ac-H3). Quantification is reported as fold change in treated cells versus DMSO control (CTR), arbitrarily set at 1 (one sample *t*-test *p*-values: * *p* < 0.05, ** *p* < 0.01, *** *p* < 0.001, **** *p* < 0.0001; *n* = 3).

To assess whether the observed antiproliferative activity of the selected compounds was a consequence of HDAC6-targeting, we examined the effect of the cell treatments on the expression levels of acetylated tubulin (Ac-Tub), a well-known HDAC6 target. LNCaP and PC-3 cells were treated with the tested compounds at their GI_50_ concentrations, and SDS-PAGE and western blot were performed. In addition, the acetylation levels of histone H3, which are mainly regulated by HDAC1, were also evaluated in order to exclude potential class I-HDAC off-target activities. We used Tubastatin-A and Geldanamycin as reference controls for cellular effects resulting from HDAC6- and Hsp90-inhibition, respectively [55,56]. The results in Figure 2 show a significant increase in α-Tubulin acetylation in LNCaP cells after treatments with compounds **8, 11** and **18**. Indeed, these compounds triggered a 2.9-, 5.1-, and 2.5-fold increase, respectively, compared to the control (DMSO). Tubastatin-A, which is a potent HDAC6 inhibitor [55], showed a 3.3-fold change under the same assay conditions. Western blot analyses led to similar results in PC-3 cells, with strong induction of α-Tubulin acetylation of 7.3-, 5.5-, and 4.9-fold, respectively, following the administration of compounds **8**, **11** and **18**, similar to the effect of Tub-A. Furthermore, no remarkable induction of H3 acetylation was observed after treatment with compound **8** in both PC-3 and LNCaP cell lines, which is informative of the putatively specific activity of the compounds on HDAC6, without affecting nuclear class I HDACs. We also evaluated the expression levels of Hsp70, a well-characterized co-chaperone of Hsp90 that is usually upregulated upon Hsp90 inhibition. As for in vitro assays, the four selected compounds did not show inhibition of Hsp90 activity. Taken together, these results corroborated the HDAC6 inhibitory activity observed in vitro and demonstrated that the selected compounds can also inhibit HDAC6 in human PCA cell lines, with compound **8** even being selective for HDAC6 versus nuclear HDACs.

### 2.4. Binding Mode of the Four Discovered Hit Compounds into HDAC6 and Their Optimization for Dual Inhibitory Activity

**Figure 3:**
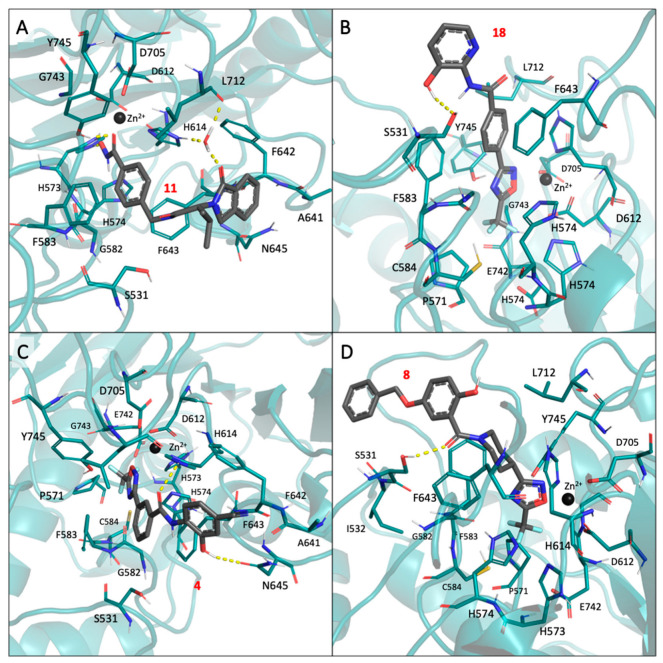
Binding mode predicted for compounds **4**, **8**, **11**, and **18** into HDAC6. In particular, (**A**) reports the docking pose of compound **11** into HDAC6 (PDB code: 5WGI) [57]. (**B**) reports the docking pose of compound **18** into HDAC6 (PDB code: 6DVL) [58]. (**C**) reports the docking pose of compound **4** into HDAC6 (PDB code: 6DVL). (**D**) reports the docking pose of compound **8** into HDAC6 (PDB code: 6DVL).

Compounds **4**, **8**, **11**, and **18** proved to be active against HDAC6, establishing favorable interactions with the residues lining the catalytic cleft of the enzyme. Specifically, the most potent HDAC6 inhibitor identified in this study, i.e., compound **11**, was predicted to fit into the HDAC6 binding site, coordinating the catalytic Zn^2+^ ion through the hydroxamate ZBG in a bidentate fashion (Figure 3A).

Moreover, the compound was predicted to form hydrogen bonds with the side chains of H573 and Y745, as well as with a conserved water molecule through the isoindolin-1-one moiety. According to docking, the aromatic linker of compound **11** fits perfectly into the catalytic tunnel of HDAC6, engaging in π-π stacking interactions with the side chains of F583 and F643.

Compared to **11**, compounds **4**, **8**, and **18**, which possess a trifluomethyloxadizole ZBG, were predicted to accommodate, on average, 1.0 Å less deep into the HDAC6 catalytic cleft (Figure 3B–D). This result, which is in line with the previous literature data [49], may arise from the stronger coordination ability of the hydroxamic acid moiety compared to TFMO. In addition, compound **18** was predicted to bind to the HDAC6 catalytic site, establishing a H bond interaction with the S531 residue (Figure 3B) and engaging in π-π stacking interactions with the side chains of F583 and F643. The amide group and hydroxyl-substituted pyridine ring were predicted to extend in the CAP region.

A similar mode of interaction was also observed for compound **4**. In particular, the phenyl ring near the TFMO ZBG made π-π stacking interactions with the side chains of F583 and F643. Moreover, the amide carbonyl oxygen established a hydrogen bond interaction with the side chain of H573, and the hydroxyl group attached to the distal phenyl ring interacted with N645.

According to docking, compound **8** exhibited a slightly different binding mode, mainly attributed to the more sterically hindered piperidine linker. Unlike compounds **4** and **18**, no π-π stacking interactions were possible for compound **8**. However, a H bond interaction was formed by its carbonyl oxygen with residue S531. Additionally, the distal 5-benzoxy-2-hydroxy-phenyl group was predicted to extend towards the cap region of HDAC6, in proximity to the side chains of H463 and P464.

Unfortunately, HDAC6 inhibitors emerging from the study did not exhibit the desired dual activity against Hsp90. This result can be partially ascribed to the fact that (i) HDAC6 and Hsp90 have significantly different binding sites; (ii) we aimed to keep the molecular weight of the compounds as low as possible to avoid molecular chimeras; and (iii) it was challenging to find compounds that recapitulated pharmacophore requirements of both targets among commercially available libraries of compounds. However, the compounds feature at least one warhead required for binding to one of the two targets, and their chemical structure is enriched with structural features found in potent inhibitors of Hsp90 and HDAC6 (see Tables S2 and S3 for general statistics), thanks to the 2D similarity screening. Therefore, making small adjustments or additions through chemical synthesis may provide the desired dual activity. For example, the cyan substituent of compound **4** could be replaced with a hydroxyl group to provide the resorcinol scaffold known to be important for binding to Hsp90 [28,59]. In addition, chlorine or isopropyl substituents can also be explored in this moiety to further improve the complementarity of the compounds towards the Hsp90 binding site [28,59]. Indeed, the isopropyl-resorcinol warhead has already been investigated also for the design of Hsp90/HDAC6 dual inhibitors providing interesting results [41]. Similarly, an additional hydroxyl group in the meta position of the hydroxyl pyridine ring of compound **18** could be introduced to mimic the resorcinol Hsp90 warhead. Further chemical explorations potentially improving the inhibitory activity towards both Hsp90 and HDAC6 might derive from the inversion of the amide moiety present in compounds **4** and **18** similarly, as previously reported [28,41,59].

As for compound **8**, a series of structural modifications could be proposed to improve HDAC6 activity while gaining inhibitory activity on Hsp90. Specifically, replacing the piperidine moiety with a phenyl ring could improve HDAC6 activity, and introducing an additional hydroxyl group in the meta position of the phenol ring could provide the resorcinol moiety important for Hsp90 activity [28,59].

Compound **11**, identified as the best candidate in the study, also exhibited significant structural similarity to several previously reported Hsp90 inhibitors (Tables S2 and S3), providing valuable insights for optimization through chemical synthesis. For example, the replacement of the isoindolin-1-one core of compound **11** with the structurally close 1,1-diketo-1,2-benzothiazol-3-one moiety, which is already present in established Hsp90 inhibitors (e.g., CHEMBL1562575 and CHEMBL1607801), could potentially result in the desired dual activity [60]. Indeed, such a substitution is not expected to impair HDAC6 activity because the group would be accommodated into the solvent-exposed cap region of the enzyme.

## 3. Discussion

Hsp90 and HDAC6 represent two established targets for the treatment of a variety of diseases, although they have primarily been studied in single-targeting approaches so far [15]. Research evidence suggests that the combined inhibition of Hsp90 and HDAC6 could yield remarkable therapeutic benefits against prostate cancer, especially in its more advanced and aggressive forms [61]. Moreover, the ability to inhibit these targets with a single molecular entity would allow circumventing issues often observed in combination therapies, in line with the polypharmacology concept [37].

With the aim of identifying candidates endowed with dual Hsp90 and HDAC6 inhibitory activity, we performed a virtual screening campaign integrating ligand- and structure-based methods on a large collection of commercial compounds. The analyses allowed the identification of a set of commercially available ligands that (i) exhibit drug-like properties; (ii) bear a functional group important for the binding to HDAC6 or Hsp90; (iii) display significant structural similarity with known inhibitors of HDAC6 and Hsp90; and (iv) are predicted to establish favorable interactions with the active sites of HDAC6 and Hsp90. The identified candidates were tested in vitro to determine HDAC6 and Hsp90 inhibitory activity and antiproliferative effects on PC-3 and LNCaP prostate cancer cells. Notably, four of the selected compounds (**4**, **8**, **11,** and **18**) exhibited both HDAC6 inhibition and antiproliferative activity in the cellular assays. Western blot analysis revealed that the effects on PCA cells observed with these candidates are most likely HDAC6-dependent. In particular, compounds **8**, **11**, and **18** increase α-Tubulin acetylation to levels comparable to the control drug Tubastatin-A in LNCaP and PC-3 cells. The effect on H3 acetylation clearly supported the hypothesis that only compound **8** is selective towards HDAC6 over nuclear HDACs. Unfortunately, none of these candidates showed inhibition of Hsp90. Since HDAC6 and Hsp90 have significantly different binding sites, this result can be partially ascribed to the fact that (i)we aimed to keep the molecular weight of the compounds as low as possible to avoid large molecular chimeras and (ii) it was challenging to find compounds that recapitulated the pharmacophore requirements of both targets among commercially available libraries. However, the identified candidates share significant similarities with Hsp90 and HDAC6 inhibitors, which can provide structural insights that can guide their optimization toward the generation of dual binders. In this regard, for example, we suggested the introduction of an additional hydroxyl group in the meta position of the hydroxyl pyridine ring of compound **18** or the replacement of the cyano substituent of compound **4** with a hydroxyl group to provide the resorcinol moiety present into potent Hsp90 inhibitors [28,59]. Moreover, we also suggested that replacing the isoindolin-1-one core of compound **11** with the 1,1-diketo-1,2-benzothiazol-3-one moiety can help in obtaining molecules that are also able to inhibit Hsp90 [60]. While limited by the commercial availability of compounds with the pharmacophoric features required for binding at both targets, the results of this study provide valuable starting points for the development of novel drug-like HDAC6/Hsp90 dual binders.

## 4. Methods

### 4.1. Molecular Modeling

#### 4.1.1. Preparation of HDAC6 and Hsp90 ChEMBL Ligands

Activity records related to Hsp90 (UniProt ID: P07900) and HDAC6 (UniProt ID: Q9UBN7) were first retrieved from the ChEMBL database (www.ebi.ac.uk/chembl/, accessed on 1 April 2020) [62], and then processed as follows. The activity records were filtered to retain only those that derived from experiments on recombinant proteins, with data reported as K_i_, K_d_, IC_50_, and EC_50_. Duplicate activity records resulting from different experiments on the same target were removed, retaining those with the best activity value. This led to 1024 and 2996 unique compounds for Hsp90 and HDAC6, respectively. Afterward, the resulting records were further processed to retain only significantly active compounds, i.e., those with an activity value (i.e., “*Standard Value*” on the ChEMBL website) below 10 µM and a “*Standard Relation*” equal to “=”.

#### 4.1.2. Protein Data Bank (PDB, www.rcsb.org/, Accessed on 1 April 2020)

X-ray complexes of Hsp90 and HDAC6 with small molecules were initially retrieved from the study of Bonanni et al. [15], and ligands were extracted in their native conformations. Then, the ligands were processed by means of the LigPrep utility (Schrödinger Suite 2020-1) to address potential atom-type issues and evaluate their most likely tautomer and ionization states at physiological pH [63]. Representative conformations of Hsp90 and HDAC6 proteins were selected, as detailed by Bonanni et al. [15] for the structure-based analyses.

#### 4.1.3. The ZINC Database Preparation

Commercial compounds available from ZINC (https://zinc15.docking.org/, accessed on 11 May 2020) [45], a publicly available database containing data related to more than 750 million purchasable molecules, were first downloaded and duplicates removed, resulting in 414,827,667 compounds. They were then filtered using the Filter software (OpenEye, Santa Fe, NM, USA, version 3.1.2.2) [64,65]. A custom filter (see Table S4) was applied to retain compounds with a chemical scaffold close to already reported drug-like molecules (see above) while ensuring a high degree of variability in their chemical structure. Subsequently, the molecules underwent a further step of filtration by using the OpenEye Toolkits 2020.1.0 [66], retaining only those bearing at least one substructure among the ZBGs and D93-binding groups previously identified.

#### 4.1.4. Two-Dimensional Ligand-Based Similarity Screening

Commercial compounds that passed the previous phases of filtering were then subjected to a series of 2D similarity screenings, which were performed using two differently designed types of fingerprints (i.e., MACCS and ECFP4) [50,51]. These fingerprints were selected for their ability to consider the similarity between molecules based on the composition in functional groups (MACCS) and structural connectivity (ECFP4). Similarity analyses were performed with default settings by using Hsp90 and HDAC6 ChEMBL molecules previously collected (see above) as queries. Commercial compounds that did not result similar to at least one Hsp90 or HDAC6 compound, according to accepted similarity thresholds (MACCS Tanimoto score > 0.8 and ECFP4 Tanimoto score > 0.3) [50] were discarded. The obtained similarity data were also taken into account during the final step of candidates’ selection.

#### 4.1.5. Structure-Based Screening

The structure-based calculations were performed using the utilities and tools implemented into Maestro (Schrödinger Suite 2020-1) [67]. In particular, commercial compounds that passed the previous filtering and similarity screening phases were prepared for the structure-based calculations by using LigPrep with default settings [63], except for the calculation of their additional metal-binding states, which was conducted to better take into account the interactions with the Zn^2+^ ion in HDAC6 binding site. The crystal structures 1UY6 [68], 2BYI [69], 2YKI [70], 2VCI [71], 3TUH [72], 4AWO [73], 4YKW [74] for Hsp90, and 5WGI [57] and 6DVL [58] for HDAC6 were selected as representative conformations in the structure-based calculations, in line with our previous studies [15]. The conformations selected for HDAC6 derived from crystallographic complexes on *Danio Rerio* proteins (i.e., Trichostatin A—PDB ligand ID: TSN) [57] and 6DVL complex (i.e., PDB ligand ID: HBG) [58], which required two of their amino acids to be mutated (i.e., N530D and N645M). Receptors’ preparation was performed with the “Protein Preparation Wizard” [75] tool available in Schrödinger Suite 2020-1 by: (i) fixing issues potentially present in the atom types of the elements in the complex; (ii) evaluating the correct tautomerization and protonation states of the residues of the proteins; (iii) adding missing hydrogens, potentially missing side chains to residues, and protein loops, and; (iv) minimizing overlapping atoms. Once prepared, all the solvent molecules derived from the crystallographic process and waters were deleted from the complexes, excluding the ones that are known to be conserved in the Hsp90 and HDAC6 binding sites [17,76]. Afterward, receptor grids, prepared for the docking calculations on the Hsp90 and HDAC6 crystal structures by means of the “Receptor Grid Generation” tool [66,77], were centered on the co-crystallized ligands within a box of 12 Å × 12 Å × 12 Å length. Default settings were used for the generation of the grids, except for structurally conserved waters, which were considered as a part of the receptor in Hsp90, and metal coordination constraints were included in HDAC6 calculations. A series of docking models were then developed with Glide [66,77] for each of the selected Hsp90 and HDAC6 representative structures and validated by redocking of the co-crystallized compounds into their parent receptor. All the validated models showed satisfactory results (i.e., Root-Mean-Square Deviation—RMSD lower than 2.0 Å). Default settings were used for the docking calculations, except for the use of constraints on the metal binding coordination for the HDAC6 complexes. Validated docking models were finally employed in the virtual screenings of the two targets. The results of the calculations were visually inspected, and the most promising candidates were selected to be purchased at Enamine (https://enamine.net/, last access 23 May 2023) and in vitro validated, as described below.

### 4.2. Experimental Methods

#### 4.2.1. Fluorimetric HDAC6 Assay

In vitro assessments of compounds on the HDAC6 enzyme were performed, as described in our previous works [12,49]. In particular, the reactions were carried out by incubating the HDAC6 recombinant enzyme, ligands, and substrate (i.e., the fluorogenic peptide from p53 residues 379–382—RHKK-Ac-AMC) with the buffer (50 mM Tris–HCl—pH 8.0, 137 mM NaCl, 2.7 mM KCl, 1 mM MgCl_2_, and 1 mg/mL BSA). All the compounds were tested with a 3-fold serial dilution in DMSO, starting from 100 μM; compound **11**, which showed high potency in the assay, was also re-tested with a 3-fold serial dilution in DMSO, starting from 1 μM. The fluorescence signal was registered after reactions completeness reading the signal at 360 nm (excitement)/460 nm (emission) light wavelength. Two micromolar volume of the reference compound was added to a solution of 16 mg/mL trypsin in 50 mM Tris–HCl, pH 8.0, 137 mM NaCl, 2.7 mM KCl, and 1 mM MgCl_2_ and used to record the fluorescence signal. The blank employed for curve fitting was evaluated on a solution of the buffer containing a concentration of 1.00 × 10^−12^ M of DMSO. IC_50_ values were calculated by using the GraphPad Prism 4 program based on a sigmoidal dose–response equation.

#### 4.2.2. Hsp90 Assay

In vitro assessments of compounds on Hsp90 were performed, as described in our previous work [30], through the competition of fluorescently labeled Geldanamycin (FITC-GM) [78]. In particular, Hsp90 assays were performed using human recombinant Hsp90α at a 30 nM concentration with His-tag, MW = 90 kDa, expressed in *E.coli* expression system in a buffer of 20 mM HEPES, pH 7.5, 50 mM NaCl, 10 mM MgCl2, 0.02% Brij 35, and Add fresh: 2 mM DTT, 0.02mg/mL BSA, 1% DMSO. First, compounds were solubilized at 10 mM in 100% DMSO, then serially diluted 1 to 3 (10 concentrations), starting from 100 μM. Then, the resulting solutions were added to the enzyme mixture and incubated for 30 min. The FITC-labeled Geldanamycin probe (used also as a positive control) was added at a 5 nM concentration to initiate the reaction and incubated for 3 hr at room temperature. Geldanamycin was used as a positive control. Fluorescence polarization was then read, and IC_50_ values and the titration curves were fit by using GraphPad Prism 8.0 software.

#### 4.2.3. Cell Lines

The human prostate cancer cell line PC-3 (ATCC Cat# CRL-1435) was grown in Ham’s F12 (Biowest, Nuaillé, France) medium, while the human prostate cancer cell line LNCaP (ATCC Cat# CRL-1740) was grown in RPMI 1640 (Biowest, Nuaillé, France) medium. Both media were supplemented with 10% Fetal Bovine Serum (FBS, Gibco, Fisher Scientific Italia, Segrate, Italy), 2 mM glutamine, 100 U/mL penicillin, and 100 μg/mL streptomycin. PC-3 and LNCaP cells were maintained at 37 °C in a humidified 5% CO_2_ atmosphere.

#### 4.2.4. Cell Viability Assays

PC-3 and LNCaP cells were seeded at a density of 4000 and 6000 cells/well into a 96-well plate, respectively, together with a calibration curve of known cell numbers. The following day, the cells were treated with the indicated test compounds at different concentrations starting at 75μM with 2-fold serial dilutions. Cell viability was evaluated after 72h by colorimetric MTT (PC-3) or PrestoBlue™ (LNCaP) cell viability reagents. A total of 0.5 mg/mL of thiazolyl blue tetrazolium bromide (MTT) (Sigma-Aldrich, St. Louis, MO, USA) in Ham’s F12 medium was added to the wells, and the plate was incubated at 37 °C for 2 h. Medium containing unconverted MTT was removed, and 100 μL of MTT solvent (4 mM HCl, 0.1% NP40, in isopropyl alcohol) was added to the wells in order to solubilize MTT crystals. After 15 min of incubation at room temperature, the absorbance values at 570 nm were obtained with a Multiskan FC Microplate Photometer (Thermo Fisher Scientific, Waltham, MA, USA). PrestoBlue reagent (#A13261, Thermo Fisher Scientific, Waltham, MA, USA) was added to the medium (1:10 *v*/*v*) and incubated for 1 h at 37 °C. Cell viability was calculated by quantifying PrestoBlue reduction by measuring the absorbance at 570 and 620 nm, according to the manufacturer’s protocol. The viability of untreated cells was arbitrarily set at 100%, and the concentration at which cellular growth is inhibited by 50% (GI_50_) was determined. Three independent experiments were performed.

#### 4.2.5. Protein Extraction and Immunoblotting

Cell treatment. PC-3 and LNCaP cells were grown in 12-well tissue-culture plates and treated with test compounds at the evaluated GI_50_ concentration for each candidate inhibitor for 24 h. DMSO was used as a negative control, while Tubastatin-A and Geldanamycin were used as positive controls for HDAC6 and Hsp90 inhibition, respectively. Vorinostat (SAHA) was used as a pan-HDACi control. Drug-treated PC-3 and LNCaP cells were harvested by trypsinization, washed with phosphate-buffered saline 1 × (PBS), and whole-cell protein extracts were prepared by lysis into 1x SDS sample buffer (25 mM Tris–HCl pH 6.8, 1.5 mM EDTA, 20% glycerol, 2% SDS, 5% b-mercaptoethenol). Protein quantitation was performed with Pierce Detergent Compatible Bradford Assay (#23246, Thermo Fisher Scientific, MA, USA), and equivalent amounts of cellular extracts were resolved by SDS-PAGE, transferred to PVDF or Nitrocellulose membrane with Trans-Blot Turbo Transfer System (Bio-Rad, Hercules, CA, USA), and immunoblotted with the following primary antibodies, diluted 1:1000 in 1× TBS with 1 mg/mL BSA: Anti-Histone H3 (#PA5-16183, Invitrogen, Thermo Fisher Scientific, MA, USA), Anti-acetylated histone H3 (#sc-518011, Santa Cruz Biotechnology, Inc., Dallas, TX, USA), Anti-Tubulin (#E-AB-20073, Elabscience, Houston, TX, USA), Anti-acetylated α-tubulin (#sc-23950, Santa Cruz), and Anti-HSP70 (#4872S, Cell Signaling Technology, Inc., Danvers, MA, USA). After incubation with secondary HRP-conjugated antibodies, Anti-mouse (#A16017, Invitrogen), and Anti-rabbit (# A16023, Invitrogen, Thermo Fisher Scientific, MA, USA), membranes were scanned with an Amersham Imager AI680 RGB (GE Healthcare Europe GmbH, Freiburg, Germany), using detection reagents Westar ηC and Supernova HRP substrates (Cyanagen, Bologna, Italy). The raw data of the Western blot experiments are available in the Supplementary Materials (Figure S4).

## 5. Conclusions

In this study, we devised and applied a virtual screening campaign on a large library of commercially available compounds, searching for molecules endowed with Hsp90/HDAC6 dual activity and antiproliferative activity against LNCaP and PC-3 prostate cancer cells. The performed analyses allowed the selection of eighteen compounds, four of which were active against the HDAC6 recombinant enzyme and also displayed antiproliferative activity against selected prostate cancer cell lines. While none of the compounds showed inhibition of Hsp90, they present low molecular size and structural features that might be optimized through organic synthesis to potentially obtain structurally novel Hsp90/HDAC6 dual binders.

## Figures and Tables

**Table 1:** In vitro inhibitory activity of the compounds against the HDAC6 recombinant enzyme (IC_50_, µM) and antiproliferative activity (GI_50_, µM) determined by cell viability assays on PC-3 and LNCaP prostate cancer cells. Tubastatin-A (Tub-A), Geldanamycin, and SAHA were used as reference compounds. Compounds shown in Figure 1 but not present in Table 1 displayed a percentage of HDAC6 inhibition lower than 50% at 20 µM. None of the compounds resulted in being active against the Hsp90 recombinant enzyme. Dose–response curves of the active compounds are reported in Figure S2 (recombinant HDAC6) and Figure S3 (cellular assays).

Compound ID	HDAC6 IC_50_ (μM)	LNCaP GI_50_ (μM)	PC-3 GI_50_ (μM)
4	17	9.5 ± 0.7	9.5 ± 0.5
8	6.5	51.6 ± 3.7	51.8 ± 0.6
11	0.005	41.3 ± 8.7	13.9 ± 2.9
18	2.7	>75	21 ± 5.4
Tubastatin-A	0.007	5.9 ± 1.5	11.1 ± 1.6
Geldanamycin		0.05 ± 0.02	0.03 ± 0.02
SAHA	0.014	1.3 ± 0.3	1.9 ± 0.2

Note: Assays on HDAC6 were performed in singlicate. GI_50_ values on PCA cells are expressed as the mean of three independent experiments ± SEM.

## Data Availability

All relevant data presented in this study are available within the article or Supplementary Material. Further inquiries can be provided by the authors upon request.

## Supplementary Materials

The following supporting information can be downloaded at: https://www.mdpi.com/article/10.3390/ph17081072/s1. Table S1: Drug-like properties predicted with QikProp for the selected compounds; Table S2: Hsp90 and HDAC6 inhibitors reported in ChEMBL that exhibited similarity to the commercial candidates showing activity on HDAC6, according to the performed ligand-based analyses; Table S3: Number of Hsp90 and HDAC6 inhibitors reported within ChEMBL with results similar to the commercial candidates resulting active against HDAC6, according to the performed ligand-based analyses; Table S4: Custom filtering criteria applied to retain compounds with a chemical scaffold closely resembling already reported drug-like molecules; Figure S1: Hsp90 and HDAC6 warheads employed within the in silico screening campaign in the initial phases of the filtering process; Figure S2: Dose–response curves of compounds 4, 8, 11, and 18 tested for in vitro inhibition of HDAC6 activity; Figure S3: Dose–response curves of the antiproliferative effects determined by PrestoBlue cell viability assay on LNCaP (panel A) and MTT cell viability assay on PC-3 (panel B) cells treated for 24 h with different doses of the synthesized compounds and of the reference standard Tubastatin-A; Figure S4: Raw data of the western blot experiments shown in Figure 2A,C.

## References

[1] Ho T.C.S., Chan A.H.Y., Ganesan A. (2020). Thirty Years of HDAC Inhibitors: 2020 Insight and Hindsight. J. Med. Chem..

[2] Cheng Y., He C., Wang M., Ma X., Mo F., Yang S., Han J., Wei X. (2019). Targeting Epigenetic Regulators for Cancer Therapy: Mechanisms and Advances in Clinical Trials. Signal Transduct. Target. Ther..

[3] Ruijter A.J.M.D., Gennip A.H.V., Caron H.N., Kemp S., Kuilenburg A.B.P.V. (2003). Histone Deacetylases (HDACs): Characterization of the Classical HDAC Family. Biochem. J..

[4] Micelli C., Rastelli G. (2015). Histone Deacetylases: Structural Determinants of Inhibitor Selectivity. Drug Discov. Today.

[5] Zhao C., Dong H., Xu Q., Zhang Y. (2020). Histone Deacetylase (HDAC) Inhibitors in Cancer: A Patent Review (2017-Present). Expert Opin. Ther. Pat..

[6] He X., Li Z., Zhuo X.-T., Hui Z., Xie T., Ye X.-Y. (2020). Novel Selective Histone Deacetylase 6 (HDAC6) Inhibitors: A Patent Review (2016–2019). Recent Pat. Anti-Cancer Drug Discov..

[7] Brindisi M., Saraswati A.P., Brogi S., Gemma S., Butini S., Campiani G. (2020). Old but Gold: Tracking the New Guise of Histone Deacetylase 6 (HDAC6) Enzyme as a Biomarker and Therapeutic Target in Rare Diseases. J. Med. Chem..

[8] Dallavalle S., Pisano C., Zunino F. (2012). Development and Therapeutic Impact of HDAC6-Selective Inhibitors. Biochem. Pharmacol..

[9] Li Y., Shin D., Kwon S.H. (2013). Histone Deacetylase 6 Plays a Role as a Distinct Regulator of Diverse Cellular Processes. FEBS J..

[10] Zhou B., Liu D., Tan Y. (2021). Role of HDAC6 and Its Selective Inhibitors in Gastrointestinal Cancer. Front. Cell Dev. Biol..

[11] Peng J., Xie F., Qin P., Liu Y., Niu H., Sun J., Xue H., Zhao Q., Liu J., Wu J. (2023). Recent Development of Selective Inhibitors Targeting the HDAC6 as Anti-Cancer Drugs: Structure, Function and Design. Bioorg. Chem..

[12] Moi D., Citarella A., Bonanni D., Pinzi L., Passarella D., Silvani A., Giannini C., Rastelli G. (2022). Synthesis of Potent and Selective HDAC6 Inhibitors Led to Unexpected Opening of a Quinazoline Ring. RSC Adv..

[13] Halsall J.A., Turner B.M. (2016). Histone Deacetylase Inhibitors for Cancer Therapy: An Evolutionarily Ancient Resistance Response May Explain Their Limited Success. BioEssays.

[14] Citarella A., Moi D., Pinzi L., Bonanni D., Rastelli G. (2021). Hydroxamic Acid Derivatives: From Synthetic Strategies to Medicinal Chemistry Applications. ACS Omega.

[15] Bonanni D., Citarella A., Moi D., Pinzi L., Bergamini E., Rastelli G. (2022). Dual Targeting Strategies on Histone Deacetylase 6 (HDAC6) and Heat Shock Protein 90 (Hsp90). Curr. Med. Chem..

[16] Wang X.-X., Wan R.-Z., Liu Z.-P. (2018). Recent Advances in the Discovery of Potent and Selective HDAC6 Inhibitors. Eur. J. Med. Chem..

[17] Hai Y., Christianson D.W. (2016). Histone Deacetylase 6 Structure and Molecular Basis of Catalysis and Inhibition. Nat. Chem. Biol..

[18] Bondarev A.D., Attwood M.M., Jonsson J., Chubarev V.N., Tarasov V.V., Schiöth H.B. (2021). Recent Developments of HDAC Inhibitors: Emerging Indications and Novel Molecules. Br. J. Clin. Pharmacol..

[19] Mottamal M., Zheng S., Huang T., Wang G. (2015). Histone Deacetylase Inhibitors in Clinical Studies as Templates for New Anticancer Agents. Molecules.

[20] Echeverría P.C., Bernthaler A., Dupuis P., Mayer B., Picard D. (2011). An Interaction Network Predicted from Public Data as a Discovery Tool: Application to the Hsp90 Molecular Chaperone Machine. PLoS ONE.

[21] Wu J., Liu T., Rios Z., Mei Q., Lin X., Cao S. (2017). Heat Shock Proteins and Cancer. Trends Pharmacol. Sci..

[22] Schopf F.H., Biebl M.M., Buchner J. (2017). The HSP90 Chaperone Machinery. Nat. Rev. Mol. Cell Biol..

[23] Pick E., Kluger Y., Giltnane J.M., Moeder C., Camp R.L., Rimm D.L., Kluger H.M. (2007). High HSP90 Expression Is Associated with Decreased Survival in Breast Cancer. Cancer Res..

[24] Ciocca D.R., Calderwood S.K. (2005). Heat Shock Proteins in Cancer: Diagnostic, Prognostic, Predictive, and Treatment Implications. Cell Stress Chaperones.

[25] Neckers L., Workman P. (2012). Hsp90 Molecular Chaperone Inhibitors: Are We There Yet?. Clin. Cancer Res..

[26] Yun C.W., Kim H.J., Lim J.H., Lee S.H. (2019). Heat Shock Proteins: Agents of Cancer Development and Therapeutic Targets in Anti-Cancer Therapy. Cells.

[27] Chai R.C., Vieusseux J.L., Lang B.J., Nguyen C.H., Kouspou M.M., Britt K.L., Price J.T. (2017). Histone Deacetylase Activity Mediates Acquired Resistance towards Structurally Diverse HSP90 Inhibitors. Mol. Oncol..

[28] Sgobba M., Rastelli G. (2009). Structure-Based and in Silico Design of Hsp90 Inhibitors. ChemMedChem.

[29] Trepel J., Mollapour M., Giaccone G., Neckers L. (2010). Targeting the Dynamic HSP90 Complex in Cancer. Nat. Rev. Cancer.

[30] Pinzi L., Foschi F., Christodoulou M.S., Passarella D., Rastelli G. (2021). Design and Synthesis of Hsp90 Inhibitors with B-Raf and PDHK1 Multi-Target Activity. ChemistryOpen.

[31] Anighoro A., Pinzi L., Marverti G., Bajorath J., Rastelli G. (2017). Heat Shock Protein 90 and Serine/Threonine Kinase B-Raf Inhibitors Have Overlapping Chemical Space. RSC Adv..

[32] Pinzi L., Benedetti R., Altucci L., Rastelli G. (2020). Design of Dual Inhibitors of Histone Deacetylase 6 and Heat Shock Protein 90. ACS Omega.

[33] Krämer O.H., Mahboobi S., Sellmer A. (2014). Drugging the HDAC6-HSP90 Interplay in Malignant Cells. Trends Pharmacol. Sci..

[34] Kovacs J.J., Murphy P.J.M., Gaillard S., Zhao X., Wu J.-T., Nicchitta C.V., Yoshida M., Toft D.O., Pratt W.B., Yao T.-P. (2005). HDAC6 Regulates Hsp90 Acetylation and Chaperone-Dependent Activation of Glucocorticoid Receptor. Mol. Cell.

[35] Kovacs J.J., Cohen T.J., Yao T.-P. (2005). Chaperoning Steroid Hormone Signaling via Reversible Acetylation. Nucl. Recept. Signal.

[36] Murphy P.J.M., Morishima Y., Kovacs J.J., Yao T.-P., Pratt W.B. (2005). Regulation of the Dynamics of Hsp90 Action on the Glucocorticoid Receptor by Acetylation/Deacetylation of the Chaperone. J. Biol. Chem..

[37] Anighoro A., Bajorath J., Rastelli G. (2014). Polypharmacology: Challenges and Opportunities in Drug Discovery. J. Med. Chem..

[38] Babcook M.A., Sramkoski R.M., Fujioka H., Daneshgari F., Almasan A., Shukla S., Nanavaty R.R., Gupta S. (2014). Combination Simvastatin and Metformin Induces G1-Phase Cell Cycle Arrest and Ripk1- and Ripk3-Dependent Necrosis in C4-2B Osseous Metastatic Castration-Resistant Prostate Cancer Cells. Cell Death Dis..

[39] Rao R., Fiskus W., Yang Y., Lee P., Joshi R., Fernandez P., Mandawat A., Atadja P., Bradner J.E., Bhalla K. (2008). HDAC6 Inhibition Enhances 17-AAG—Mediated Abrogation of Hsp90 Chaperone Function in Human Leukemia Cells. Blood.

[40] Ai J., Wang Y., Dar J.A., Liu J., Liu L., Nelson J.B., Wang Z. (2009). HDAC6 Regulates Androgen Receptor Hypersensitivity and Nuclear Localization via Modulating Hsp90 Acetylation in Castration-Resistant Prostate Cancer. Mol. Endocrinol..

[41] Wu T.-Y., Chen M., Chen I.-C., Chen Y.-J., Chen C.-Y., Wang C.-H., Cheng J.-J., Nepali K., Chuang K.-H., Liou J.-P. (2023). Rational Design of Synthetically Tractable HDAC6/HSP90 Dual Inhibitors to Destroy Immune-Suppressive Tumor Microenvironment. J. Adv. Res..

[42] Warr W.A., Nicklaus M.C., Nicolaou C.A., Rarey M. (2022). Exploration of Ultralarge Compound Collections for Drug Discovery. J. Chem. Inf. Model..

[43] Lyu J., Wang S., Balius T.E., Singh I., Levit A., Moroz Y.S., O’Meara M.J., Che T., Algaa E., Tolmachova K. (2019). Ultra-Large Library Docking for Discovering New Chemotypes. Nature.

[44] Rastelli G., Pinzi L. (2015). Computational Polypharmacology Comes of Age. Front. Pharmacol..

[45] Sterling T., Irwin J.J. (2015). ZINC 15—Ligand Discovery for Everyone. J. Chem. Inf. Model..

[46] Zhang L., Zhang J., Jiang Q., Zhang L., Song W. (2018). Zinc Binding Groups for Histone Deacetylase Inhibitors. J. Enzym. Inhib. Med. Chem..

[47] Magwenyane A.M., Mhlongo N.N., Lawal M.M., Amoako D.G., Somboro A.M., Sosibo S.C., Shunmugam L., Khan R.B., Kumalo H.M. (2020). Understanding the Hsp90 N-Terminal Dynamics: Structural and Molecular Insights into the Therapeutic Activities of Anticancer Inhibitors Radicicol (RD) and Radicicol Derivative (NVP-YUA922). Molecules.

[48] Porter N.J., Shen S., Barinka C., Kozikowski A.P., Christianson D.W. (2018). Molecular Basis for the Selective Inhibition of Histone Deacetylase 6 by a Mercaptoacetamide Inhibitor. ACS Med. Chem. Lett..

[49] Moi D., Bonanni D., Belluti S., Linciano P., Citarella A., Franchini S., Sorbi C., Imbriano C., Pinzi L., Rastelli G. (2023). Discovery of Potent Pyrrolo-Pyrimidine and Purine HDAC Inhibitors for the Treatment of Advanced Prostate Cancer. Eur. J. Med. Chem..

[50] Jasial S., Hu Y., Vogt M., Bajorath J. (2016). Activity-Relevant Similarity Values for Fingerprints and Implications for Similarity Searching. F1000Research.

[51] Rogers D., Hahn M. (2010). Extended-Connectivity Fingerprints. J. Chem. Inf. Model..

[52] (2020). Schrödinger Release 2020-1: QikProp, Schrödinger, LLC, New York, NY, USA. https://www.schrodinger.com/.

[53] Horoszewicz J.S., Leong S.S., Kawinski E., Karr J.P., Rosenthal H., Chu T.M., Mirand E.A., Murphy G.P. (1983). LNCaP Model of Human Prostatic Carcinoma. Cancer Res..

[54] Namekawa T., Ikeda K., Horie-Inoue K., Inoue S. (2019). Application of Prostate Cancer Models for Preclinical Study: Advantages and Limitations of Cell Lines, Patient-Derived Xenografts, and Three-Dimensional Culture of Patient-Derived Cells. Cells.

[55] Shen S., Svoboda M., Zhang G., Cavasin M.A., Motlova L., McKinsey T.A., Eubanks J.H., Bařinka C., Kozikowski A.P. (2020). Structural and in Vivo Characterization of Tubastatin A, a Widely Used Histone Deacetylase 6 Inhibitor. ACS Med. Chem. Lett..

[56] Stebbins C.E., Russo A.A., Schneider C., Rosen N., Hartl F.U., Pavletich N.P. (1997). Crystal Structure of an Hsp90-Geldanamycin Complex: Targeting of a Protein Chaperone by an Antitumor Agent. Cell.

[57] Porter N.J., Mahendran A., Breslow R., Christianson D.W. (2017). Unusual Zinc-Binding Mode of HDAC6-Selective Hydroxamate Inhibitors. Proc. Natl. Acad. Sci. USA.

[58] Porter N.J., Osko J.D., Diedrich D., Kurz T., Hooker J.M., Hansen F.K., Christianson D.W. (2018). Histone Deacetylase 6-Selective Inhibitors and the Influence of Capping Groups on Hydroxamate-Zinc Denticity. J. Med. Chem..

[59] Dymock B.W., Barril X., Brough P.A., Cansfield J.E., Massey A., McDonald E., Hubbard R.E., Surgenor A., Roughley S.D., Webb P. (2005). Novel, Potent Small-Molecule Inhibitors of the Molecular Chaperone Hsp90 Discovered through Structure-Based Design. J. Med. Chem..

[60] CHEMBL1201862 Document Report Card. https://www.ebi.ac.uk/chembl/document_report_card/CHEMBL1201862/.

[61] George P., Bali P., Annavarapu S., Scuto A., Fiskus W., Guo F., Sigua C., Sondarva G., Moscinski L., Atadja P. (2005). Combination of the Histone Deacetylase Inhibitor LBH589 and the Hsp90 Inhibitor 17-AAG Is Highly Active against Human CML-BC Cells and AML Cells with Activating Mutation of FLT-3. Blood.

[62] Gaulton A., Hersey A., Nowotka M., Bento A.P., Chambers J., Mendez D., Mutowo P., Atkinson F., Bellis L.J., Cibrián-Uhalte E. (2017). The ChEMBL Database in 2017. Nucleic Acids Res..

[63] (2020). Schrödinger Release 2020-1: LigPrep, Schrödinger, LLC, New York, NY, USA. https://www.schrodinger.com/.

[64] Hawkins P.C.D., Skillman A.G., Warren G.L., Ellingson B.A., Stahl M.T. (2010). Conformer Generation with OMEGA: Algorithm and Validation Using High Quality Structures from the Protein Databank and Cambridge Structural Database. J. Chem. Inf. Model..

[65] OMEGA 3.0.1.2: OpenEye Scientific Software, Santa Fe, NM, USA. http://www.eyesopen.com.

[66] (2020). OpenEye Python Toolkits 2020.1.0, OpenEye Scientific Software, Santa Fe, NM, USA. http://www.eyesopen.com.

[67] (2020). Schrödinger Release 2020-1: Maestro, Schrödinger, LLC, New York, NY, USA. https://www.schrodinger.com/.

[68] Wright L., Barril X., Dymock B., Sheridan L., Surgenor A., Beswick M., Drysdale M., Collier A., Massey A., Davies N. (2004). Structure-Activity Relationships in Purine-Based Inhibitor Binding to HSP90 Isoforms. Chem. Biol..

[69] Brough P.A., Barril X., Beswick M., Dymock B.W., Drysdale M.J., Wright L., Grant K., Massey A., Surgenor A., Workman P. (2005). 3-(5-Chloro-2,4-Dihydroxyphenyl)-Pyrazole-4-Carboxamides as Inhibitors of the Hsp90 Molecular Chaperone. Bioorganic Med. Chem. Lett..

[70] Vallée F., Carrez C., Pilorge F., Dupuy A., Parent A., Bertin L., Thompson F., Ferrari P., Fassy F., Lamberton A. (2011). Tricyclic Series of Heat Shock Protein 90 (Hsp90) Inhibitors Part I: Discovery of Tricyclic Imidazo[4,5-c]Pyridines as Potent Inhibitors of the Hsp90 Molecular Chaperone. J. Med. Chem..

[71] Brough P.A., Aherne W., Barril X., Borgognoni J., Boxall K., Cansfield J.E., Cheung K.-M.J., Collins I., Davies N.G.M., Drysdale M.J. (2008). 4,5-Diarylisoxazole Hsp90 Chaperone Inhibitors: Potential Therapeutic Agents for the Treatment of Cancer. J. Med. Chem..

[72] Ying W. Crystal Structure of the N-Terminal Domain of an HSP90 in the Presence of an the Inhibitor. https://www.rcsb.org/structure/3TUH.

[73] Bussenius J., Blazey C.M., Aay N., Anand N.K., Arcalas A., Baik T., Bowles O.J., Buhr C.A., Costanzo S., Curtis J.K. (2012). Discovery of XL888: A Novel Tropane-Derived Small Molecule Inhibitor of HSP90. Bioorganic Med. Chem. Lett..

[74] Kang Y.N., Stuckey J.A. Structure of Heat Shock Protein 90 Bound to CS312. https://www.rcsb.org/structure/4YKW.

[75] Madhavi Sastry G., Adzhigirey M., Day T., Annabhimoju R., Sherman W. (2013). Protein and Ligand Preparation: Parameters, Protocols, and Influence on Virtual Screening Enrichments. J. Comput. Aided Mol. Des..

[76] Yan A., Grant G.H., Graham Richards W. (2008). Dynamics of Conserved Waters in Human Hsp90: Implications for Drug Design. J. R. Soc. Interface..

[77] Friesner R.A., Banks J.L., Murphy R.B., Halgren T.A., Klicic J.J., Mainz D.T., Repasky M.P., Knoll E.H., Shelley M., Perry J.K. (2004). Glide: A New Approach for Rapid, Accurate Docking and Scoring. 1. Method and Assessment of Docking Accuracy. J. Med. Chem..

[78] Howes R., Barril X., Dymock B.W., Grant K., Northfield C.J., Robertson A.G.S., Surgenor A., Wayne J., Wright L., James K. (2006). A Fluorescence Polarization Assay for Inhibitors of Hsp90. Anal. Biochem..

