# Supplementary material for: Searching for Novel HDAC6/Hsp90 Dual Inhibitors with Anti-Prostate Cancer Activity: In Silico Screening and In Vitro Evaluation
Source: Pharmaceuticals (Basel). 2024 Aug 15;17(8):1072. doi: 10.3390/ph17081072 (PMC11357446; doi:10.3390/ph17081072)

SUPPORTING INFORMATION

Searching for novel HDAC6/Hsp90 dual inhibitors with anti-prostate cancer activity: in silico screening and in vitro evaluation

*Luca Pinzi, Silvia Belluti, Isabella Piccinini, Carol Imbriano, Giulio Rastelli**

Department of Life Sciences, University of Modena and Reggio Emilia, Via Giuseppe Campi 103, 41125, Modena, Italy.

Corresponding Author
Giulio Rastelli
University of Modena and Reggio Emilia
Via Campi 103, 41125 Modena, Italy
Phone: +39 059 2058564
Email: giulio.rastelli@unimore.it

TABLE OF CONTENTS

Tables

Table S1…………………………………………………………………………………… S3
Table S2…………………………………………………………………………………… S4
Table S3…………………………………………………………………………………… S5
Table S4…………………………………………………………………………………… S6

Figures

Figure S1 …………………………………………………………………………..…… S7
Figure S2 ……………………………………………………………………………... S8
Figure S3 ……………………………………………………………………………... S9
Figure S4……………………………………………………………………………...S10

Table S1: Drug-like properties predicted with QikProp for the selected compounds. In particular, CNS is the predicted central nervous system activity on a –2 (inactive) to +2 (active) scale; QPlogS is the predicted aqueous solubility (mol*dm^{-3}) defined as the concentration of the solute in a saturated solution that is in equilibrium with the crystalline solid; QPPCaco is the predicted apparent Caco-2 cell permeability (nm*sec^{-1}); PSA is the Van der Waals surface area of polar nitrogen and oxygen atoms and carbonyl carbon atoms; RuleOfFive is number of violations of Lipinski's rule of five (i.e., mol_MW < 500, QplogPo/w < 5, donorHB ≤ 5, accptHB ≤ 10). RuleOfThree is the number of violations of Jorgensen's rule of three (i.e., QPlogS > -5.7, QPPCaco > 22 nm/s, # Primary Metabolites < 7). The compounds were also profiled for potential PAINS by using the http://zinc15.docking.org/patterns/home/ online tool. None of the compounds was predicted to be a PAINS.

Compound ID	CNS	QPlogS	QPPCaco	PSA	RuleOfFive	RuleOfThree
**1**	-2	-2.871	19.983	150.071	0	1
**2**	-2	-2.665	13.883	157.421	1	1
**3**	-1	-5.303	436.732	93.157	0	0
**4**	-2	-5.930	86.154	120.545	0	1
**5**	-1	-5.461	283.960	107.396	0	0
**6**	0	-4.368	341.909	100.017	0	0
**7**	0	-4.319	467.879	99.069	0	0
**8**	-1	-6.356	535.791	95.644	0	1
**9**	-2	-3.615	312.488	97.390	0	0
**10**	-1	-4.746	708.539	76.493	0	0
**11**	-2	-3.619	137.020	123.256	0	0
**12**	-2	-4.639	38.462	131.257	0	0
**13**	0	-5.728	370.575	94.703	0	1
**14**	-1	-5.016	370.779	94.702	0	0
**15**	-2	-3.761	210.473	121.746	0	0
**16**	-2	-4.548	40.888	128.679	0	1
**17**	-1	-5.459	391.814	94.692	0	0
**18**	-1	-4.556	255.616	107.721	0	0

Table S2: Hsp90 and HDAC6 inhibitors reported in ChEMBL that exhibited similarity to the commercial candidates showing activity on HDAC6, according to the performed ligand-based analyses. Only similarity records above thresholds (MACCS*fp* Tanimoto index ≥ 0.8 and ECFP4*fp* Tanimoto index ≥ 0.3) with respect to Hsp90 and HDAC6 ligands are reported.

Compound ID	*HDAC6 ChEMBL ID*	*MACCSfp similarity score*	*ECFP4fp similarity score*	*Standard Type*	*Standard Value*	*DOC ChEMBL ID*
**4**	CHEMBL3692609	0,82	0,85	IC50	6100	CHEMBL3638656
**4**	CHEMBL3692624	0,82	0,71	IC50	2500	CHEMBL3638656
**4**	CHEMBL3692603	0,84	0,71	IC50	8300	CHEMBL3638656
**4**	CHEMBL3692610	0,80	0,71	IC50	8600	CHEMBL3638656
**4**	CHEMBL3692584	0,81	0,70	IC50	4800	CHEMBL3638656
**4**	CHEMBL3692583	0,80	0,70	IC50	2100	CHEMBL3638656
**4**	CHEMBL3692597	0,81	0,48	IC50	7100	CHEMBL3638656
**8**	CHEMBL3692681	0,83	0,42	IC50	3200	CHEMBL3638656
**8**	CHEMBL3692663	0,80	0,42	IC50	3000	CHEMBL3638656
**8**	CHEMBL3688148	0,85	0,40	IC50	5500	CHEMBL3638656
**8**	CHEMBL3692580	0,84	0,40	IC50	5700	CHEMBL3638656
**8**	CHEMBL3692680	0,80	0,35	IC50	6700	CHEMBL3638656
**11**	CHEMBL481719	0,81	0,49	IC50	110	CHEMBL1143301
**11**	CHEMBL247217	0,80	0,49	IC50	230	CHEMBL1140672
**11**	CHEMBL197774	0,82	0,43	IC50	709	CHEMBL1142838
**11**	CHEMBL247218	0,81	0,43	IC50	990	CHEMBL1140672
**11**	CHEMBL4292944	0,80	0,42	IC50	13,6	CHEMBL4270518
**11**	CHEMBL4284297	0,82	0,41	IC50	3,78	CHEMBL4270518
**11**	CHEMBL3971436	0,82	0,35	IC50	4,08	CHEMBL3886619
**11**	CHEMBL3926575	0,84	0,33	IC50	47	CHEMBL3886619
**11**	CHEMBL4468860	0,82	0,33	IC50	1995,26	CHEMBL4385610
**11**	CHEMBL4782478	0,8	0,46	IC50	33,3	CHEMBL4680193
**18**	CHEMBL3692584	0,85	0,85	IC50	4800	CHEMBL3638656
**18**	CHEMBL3692583	0,83	0,85	IC50	2100	CHEMBL3638656
**18**	CHEMBL3692603	0,87	0,79	IC50	8300	CHEMBL3638656
**18**	CHEMBL3692610	0,83	0,79	IC50	8600	CHEMBL3638656
**18**	CHEMBL3692604	0,82	0,79	IC50	5300	CHEMBL3638656
**18**	CHEMBL3692624	0,85	0,74	IC50	2500	CHEMBL3638656
**18**	CHEMBL3692609	0,82	0,72	IC50	6100	CHEMBL3638656
**18**	CHEMBL3692597	0,81	0,56	IC50	7100	CHEMBL3638656

Compound ID	*Hsp90 ChEMBL ID*	*MACCSfp similarity score*	*ECFP4fp similarity score*	*Standard Type*	*Standard Value (nM)*	*DOC ChEMBL ID*
**8**	CHEMBL3600506	0,81	0,40	IC50	45,00	CHEMBL3600311
**8**	CHEMBL4060353	0,80	0,38	Ki	32,00	CHEMBL4007418
**8**	CHEMBL4096929	0,81	0,33	IC50	39,00	CHEMBL4041474
**8**	CHEMBL3600434	0,80	0,31	IC50	66,00	CHEMBL3600311
**11**	CHEMBL4782478	0,80	0,46	IC50	66,00	CHEMBL4680193
**11**	CHEMBL4245766	0,80	0,38	IC50	85,70	CHEMBL4229357

Table S3: Number of Hsp90 and HDAC6 inhibitors reported within ChEMBL that resulted similar to the commercial candidates resulting active against HDAC6, according to the performed ligand-based analyses. Only similarity records above thresholds (MACCS*fp* Tanimoto index ≥ 0.8 and/or ECFP4*fp* Tanimoto index ≥ 0.3) with respect to Hsp90 and HDAC6 ligands are considered. Compound **11** is the only candidate that resulted similar to known Hsp90/HDAC6 dual inhibitors reported in ChEMBL.

Compound ID	***HDAC6 - number of similar ligands***			***Hsp90 - number of similar ligands***		
	MACCSfp	***ECFP4fp***	***MACCSfp & ECFP4fp***	***MACCSfp***	***ECFP4fp***	***MACCSfp & ECFP4fp***
**4**	7	861	7	0	639	0
**8**	6	1286	5	4	517	4
**11**	10	1773	10	1	177	1
**18**	8	1151	8	0	672	0

Table S4: Custom filtering criteria applied to retain compounds with a chemical scaffold closely resembling already reported drug-like molecules.

Descriptor	***Value***
Molecular Weight	Range: 130 to 780
Number of heavy atoms	Range: 9 to 55
Number of carbons	Range: 3 to 41
Number of heteroatoms	Range: 1 to 20
Number of ring systems	Range: 2 to 10
Number of atoms the ring systems	Range: 0 to 20
Number of H-bond donors	Range: 0 to 20
Number of H-bond acceptors	Range: 0 to 20
Number of chiral centers	Range: 0 to 21
Metals	Sc, Ti, V, Cr, Mn, Fe, Co, Ni, Cu, Zn, Y, Zr, Nb, Mo, Tc, Ru, Rh, Pd, Ag, Cd

Figure S1: Hsp90 and HDAC6 warheads employed within the in silico screening campaign in the initial phases of the filtering process.

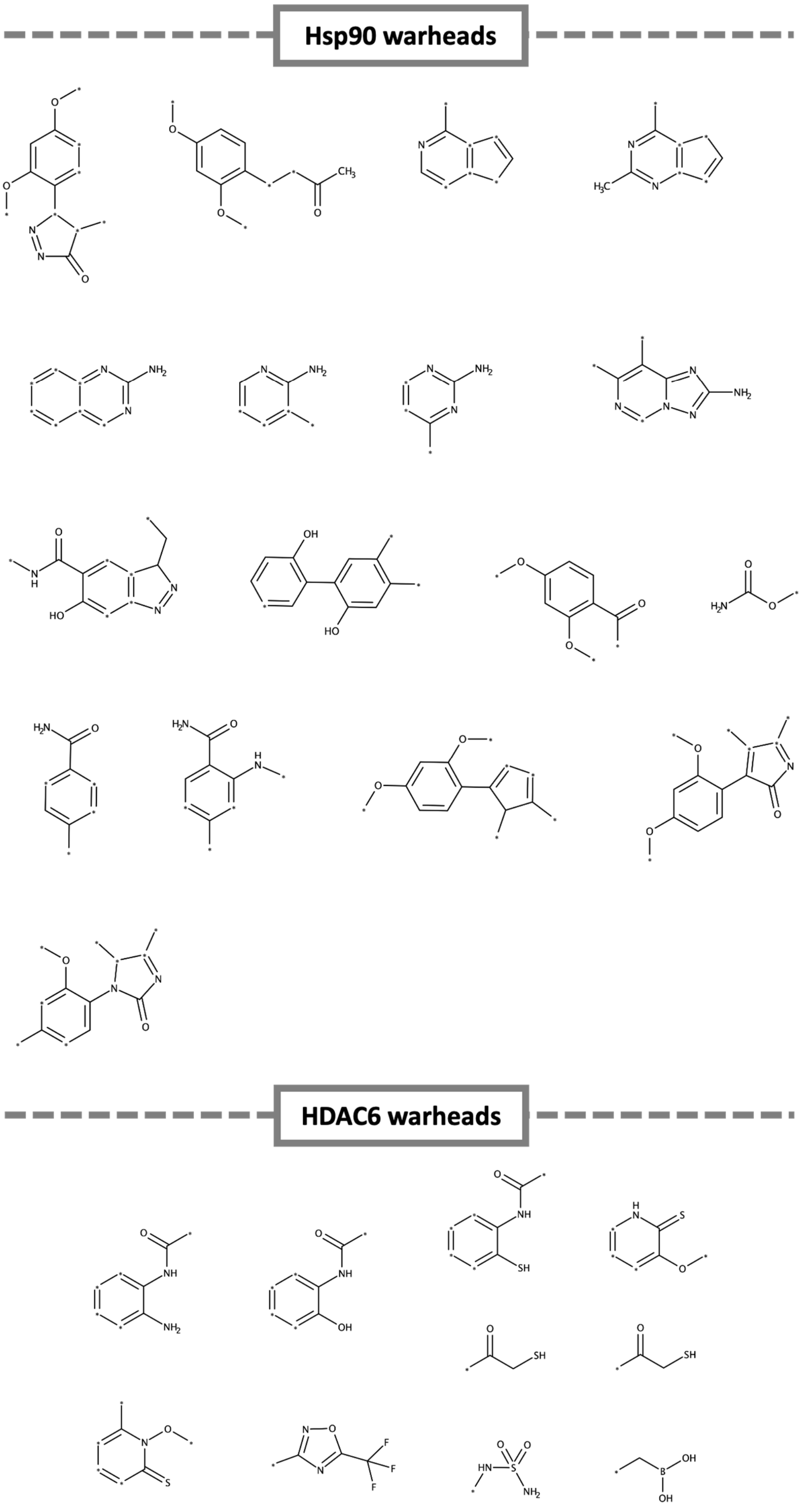

Figure S2. Dose-response curves of compounds **4**, **8**, **11**, **18** tested for in vitro inhibition of HDAC6 activity. All the compounds were tested in singlet 10-dose IC_{50} mode with 3-fold serial dilution starting from 100 μM (panel A). The most active compound **11** was also tested in a 10-dose IC_{50} with 3-fold serial dilution starting from 1 μM (panel B), as for the references Tubastatin A and SAHA (Vorinostat) . The dose-response curve of Tubastatin A and SAHA are reported as a control (panel C). IC_{50} values were calculated using the GraphPad Prism4 program based on a sigmoidal dose-response equation.

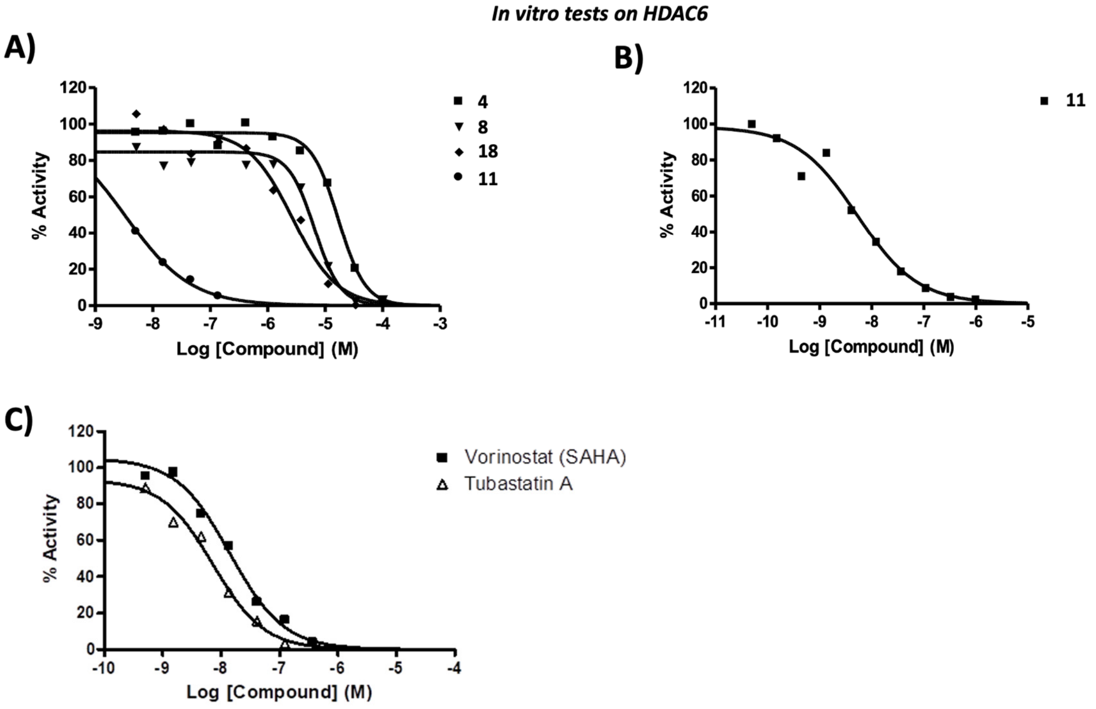

Figure S3: Dose-response curves of the anti-proliferative effects determined by PrestoBlue cell viability assay on LNCaP (panel A), and MTT cell viability assay on PC-3 (panel B) cells treated for 24h with different doses of the synthesized compounds and of the reference standard Tubastatin A.

A

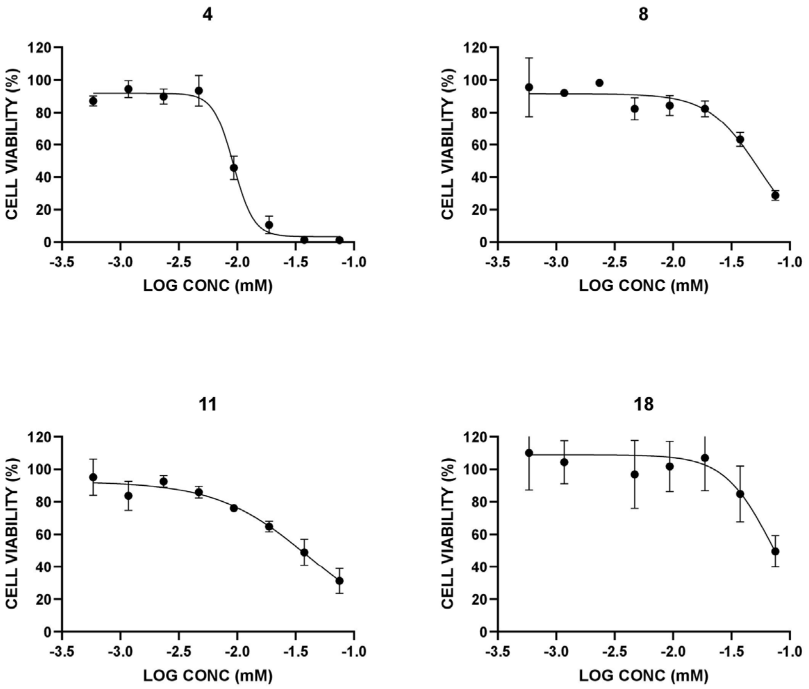

B

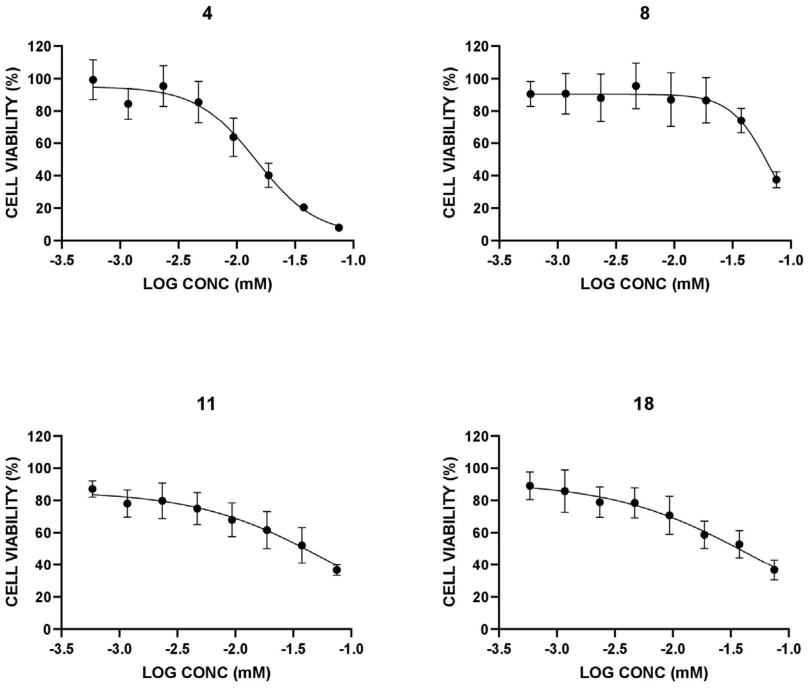

Figure S4: Raw data of the Western blot experiments shown in Figure 2A (panel A) and Figure 2C (panel B).

A

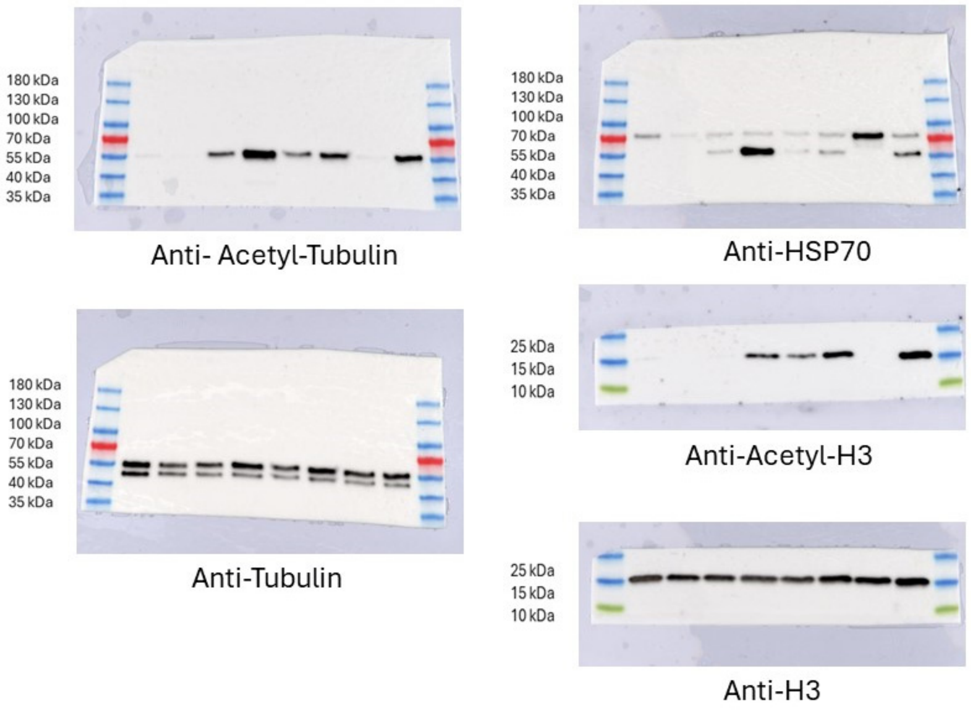

B

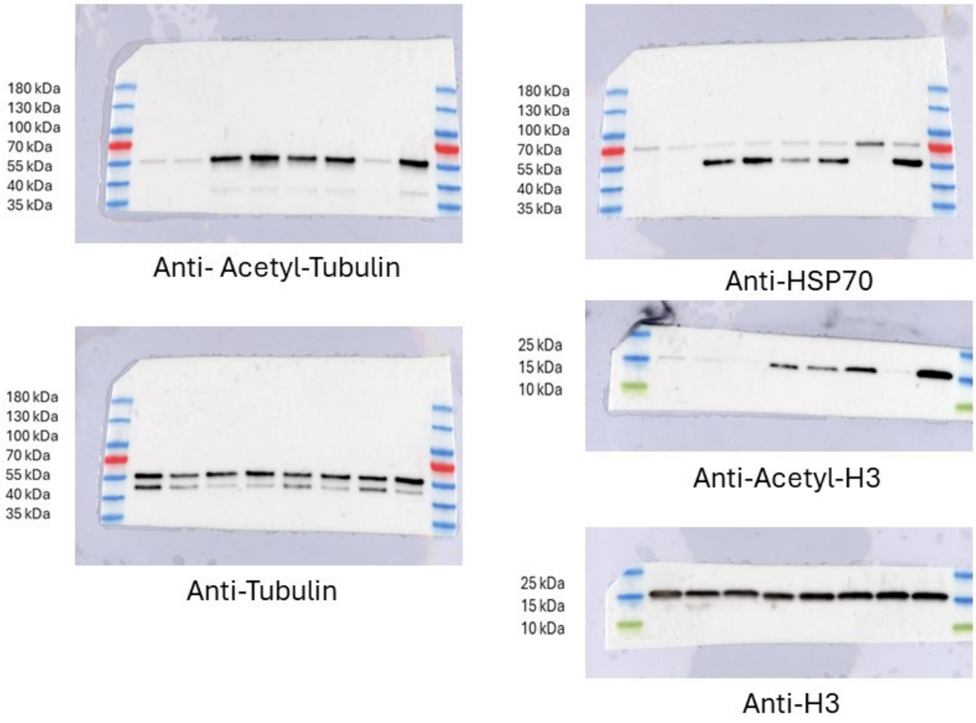

Supplement: Supplementary file 1 [file pharmaceuticals-17-01072-s001.zip › pharmaceuticals-3149290-supplementary.pdf]
